# Prolactin gene polymorphisms and associations with reproductive traits in Indonesian local ducks

**DOI:** 10.14202/vetworld.2020.2301-2311

**Published:** 2020-11-03

**Authors:** Dattadewi Purwantini, R. Singgih Sugeng Santosa, Setya Agus Santosa, Agus Susanto, Dewi Puspita Candrasari, Ismoyowati Ismoyowati

**Affiliations:** Faculty of Animal Science, University of Jenderal Soedirman, Indonesia

**Keywords:** egg production, Magelang, Prolactin gene, reciprocal mating, single nucleotide polymorphisms, Tegal

## Abstract

**Background and Aim:**

Reproductive traits play an important role in population increases and the egg production (EP) abilities of Indonesian local ducks (ILD). The prolactin (*PRL*) gene is a single chain polypeptide hormone belonging to a family of growth hormone genes that are mainly synthesized in the anterior pituitary gland in all vertebrates. It has a significant effect on reproductive traits and EP. Single nucleotide polymorphisms (SNPs) present in *PRL* are a useful molecular marker for EP. This study aimed to identify the *PRL* polymorphisms based on these SNPs and to uncover the associations with reproductive traits in ILD.

**Materials and Methods:**

A total of 280 ILDs consisting of Tegal and Magelang (F0) ducks and their reciprocal crosses, namely, Gallang (F1) and Maggal (F1), were maintained and specific variables were recorded, that is, age at first egg, body weight at first egg, first egg weight, and EP, for 90 days. Allele and genotype frequencies were used to determine the Hardy-Weinberg (H-W) equilibrium. The association between the SNP genotypes of *PRL* and reproductive traits was analyzed using one-way analysis of variance, following the GLM procedure of SAS. The genotypic effects on the reproductive traits were determined using regression analysis.

**Results:**

This study successfully amplified a polymerase chain reaction product of 190 bp, which was used to identify the SNP. Results indicated that *PRL* in ILDs is polymorphic. A SNP was found at position 164 nt (c.164G >A), consisting of three different genotypes, namely, GG, GA, and AA. The genotypes of Tegal and Magelang (F0), and Gallang (F1) populations were not in H-W equilibrium. The Maggal population (F1) was in H-W equilibrium. Significant associations were detected between the genotypes and EP in all ILDs (p<0.01), following a regression line of y=2.337x+64.605, with a determination coefficient of 0.0188 (r=0.14).

**Conclusion:**

*PRL* can be recommended as a candidate gene for reproductive traits in ILD, especially EP.

## Introduction

**Figure-1:**
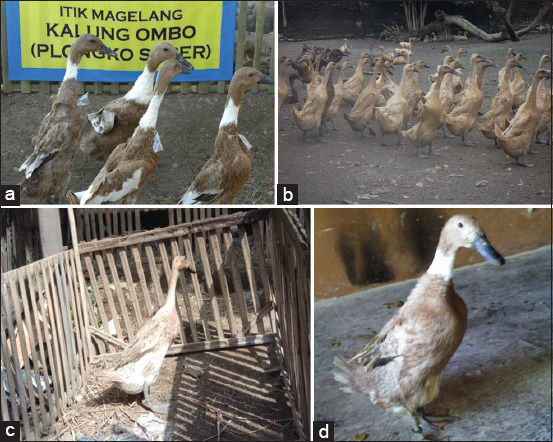
Indonesian local ducks (a). Magelang duck, (b). Tegal ducks, (c). Gallang duck, (d). Magal duck.

Indonesian local duck (ILD) germ plasma is an important Indonesian source of protein (eggs and meat) and income for rural communities, contributing 14.72% or 290.10 thousand tons of the national egg demand [1]. Another advantage of ducks compared to other birds is their high adaptability to the novel environments, simplifying production in almost all regions of Indonesia [2]. ILD’s reared by the majority of rural communities are mostly Tegal and Magelang ducks, the growing types in the Central Java Province, which exhibit improved egg production (EP) [3]. Duck farming in Indonesia is a government program that aims to increase its population and production capabilities by improving production and reproduction. Reciprocal crossing between Tegal and Magelang ducks can be performed to obtain offspring with superior reproductive ability and increased EP percentages. The crossing of Tegal drakes with Magelang ducks is called Gallang. The crossing of Magelang drakes with Tegal ducks is called Maggal [4]. Crossings are done as a production strategy to exploit the resultant hybrid vigor (heterosis) in an effort to increase livestock productivity. The Indonesia local ducks and the reciprocal crossings are presented in Figure-1.

Population genetic diversity and polymorphisms form the basis of breeding application technologies in livestock utilization. The phenotypic variation of a population expressed as a quantitative characteristic is the reflection of the population’s genetic makeup. *PRL* is a single chain polypeptide hormone belonging to a family of growth hormone genes that are mainly synthesized in the anterior pituitary gland in all vertebrates [5,6]. Specifically, by lactotrophic cells produced in pituitary gonadotropins, promoting gonad growth, steroidogenesis, gametogenesis, and ovulation in all vertebrates [7]. Prolactin (*PRL*) has a significant effect on reproductive traits and EP [6] related to the post-mating phase of the reproductive cycle [7]. Expression of *PRL* is regulated by sequences in the 5′-flanking region through binding to specific transcription factors [8]. Other proteins are also and important in regulating *PRL* expression through certain promoter binding sites.

The *PRL* gene is associated with the reproductive traits of ducks, including egg shell strength, EP, and egg weight, the genes of which are located in exon 2, 4, and 5, respectively [6]. Chicken *PRL* single nucleotide polymorphisms (SNPs) are located in exon 5, are significantly associated with parent behavior and EP [9], and are useful as molecular markers for EP in chickens [8,10]. However, research on the association of *PRL* SNPs with reproductive traits in ILD populations, especially Tegal and Magelang (F0), and the reciprocal crosses (F1), has not been done.

This study aimed to identify the *PRL* SNP and its association with the reproductive traits of Indonesia’s local duck populations. This research intends to obtain genetic markers based on *PRL* SNPs that can be used as a basis for early selection of local ducks and to provide important references for the improvement of biomolecular-based genetic quality in ILD breeding programs.

## Materials and Methods

### Ethical approval

The experimental protocols were approved by the Animal Ethics Committee, Jenderal Soedirman University No. 158/UN.23/14/PN.01.00/2019.

### Study period, location, Animals and sampling

The study included 280 female Tegal (F0) and Magelang (F0) ducks, and their reciprocal crosses, namely, Gallang (F1) and Maggal (F1) each with equal numbers (70 animals) and of the same age (4-months-old) were collected from March 2019 to February 2020. The population was reared at an experimental farm of Faculty of Animal Science, University of Jenderal Soedirman, Indonesia, under uniform management regarding ethical animal standards. The pen mating model with an open-sided house system was equipped with individual nesting chambers of 1.5×1.5 m/pen. Forty pens were used in the study, each of which contained one male and seven females. Laying ducks were given (Table-1) a 160 g/head feed per day and drinking water was available *ad libitum*. The reproductive traits observed included age at first egg (AFE), body weight at first egg (BW), first egg weight (FEW), and EP for 90 days. The AFE was obtained from individual records of the hatching dates up until the first egg laying. The duck body weights were measured when the first egg was laid to obtain BW. Eggs were also weighed to obtain the FEW. The EP was obtained by adding the number of eggs each duck laid within 90 days from the 1^st^ day of egg laying.

**Table-1:** Composition and nutrient content of laying ducks feed.

Feed stuffs	Feed content (%)
Corn	35.0
Fishmeal	10.0
Rice bran	45.0
Meat bone meal	7.0
Milled corn cobs	2.0
Premix	1.0
Total	100
Feed nutrient content	
Crude protein (%)	16.95
ME (kcal/kg)	2.876
Crude fiber (%)	7.86
Crude fat (%)	8.07
Ca (%)	0.56
P (%)	0.97

Source: Calculation based on NRC (2004) and proximate (2019). ME=Metabolisable energy, Ca=Calcium, P=Phosphor


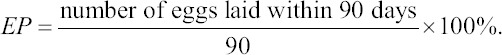


### DNA isolation, primer design, and polymerase chain reaction (PCR) amplification

DNA genomes were isolated from 280 4-month-old local ducks by drawing blood samples and using the DNA Isolation Kit High Pure PCR template preparation (Geneaid, Taiwan), according to the manufacturer’s protocol. Isolated DNA was used as a PCR template without the purification process. The primary design of oligonucleotides specific for the *PRL* gene was based on data from GenBank (JQ677091.1, 2012), namely, *Anas platyrhynchos*. Primer pairs were identified in the conserved area and analyzed using Oligoprimer Design Software with an Online Primer3 program (http://www-genome.wi.mit. edu/cgi.bin/ primr3.cgi/results_from-primer3). The primer pairs used were forward *PRL*-AnasPF primers, namely: L 2294 5’- ATAACGCCTCTCCTTGCTGA-3’, and reverse *PRL*-AnasPR, namely: H 2463 5’- TTTTCCTCCCCCTCTGTCT-3’. A PCR product of 190 bp was amplified, sequenced, and analyzed. The amplification process was performed through PCR using the GeneAmpR PCR system thermocycler 2400 (Perkin Elmer). The PCR reagent mixture consisted of 12.5 μL KAPA (PCR Kit), 1μL (10 pmol/μL) of each primer, 9.5 μL dH_2_O free nuclease, and 1 μL DNA template, with a total volume of 25 μL. The PCR cycle consisted of four stages, namely: Pre-denaturation at 94°C for 5 min, denaturation at 94°C for 30 s, annealing at 55°C for 45 s, elongation (extension) at 72°C for 1 min, and post elongation at 72°C for 5 min, which was repeated for 35 cycles. The PCR product was separated by electrophoresis in low melting agarose gel 1% and 1×TBE buffer in the Submarine Electrophoresis device (Hoefer, USA) at 50 V for 15 min. The PCR products were then visualized by UV illumination.

### DNA sequencing

PCR products were sequenced using the same primers (*PRL*-AnasPF and *PRL*-AnasPR). The sequencing process was performed by PT Genetics Science Indonesia. The sequencing product was read using Sequence Scanner v1.0 software, in the form of an electropherogram consisting of nucleotide sequences from the *PRL* gene samples of Tegal and Magelang ducks (F0), and their reciprocal crosses (F1). Each nucleotide produces peaks with different colors on an electropherogram, that is, adenine (A)=Green, guanine (G)=Black, cytosine (C)=Blue, and thymine (T)=Red.

### Polymorphism identification and genotyping

*PRL* gene polymorphisms were determined using the BioEdit v7.2.0 program (https://bioedit.software.informer.com/7.2/) by aligning the sequencing results to those obtained from GenBank (JQ677091.1) using the ClustalW (accessory application) option. Alignment results were viewed on the electropherogram so that the SNP is obtained at a certain position, which was then used to determine the genotype of each individual, for example, on SNP c.164G >A, alleles G, and A were observed so that there were three pairs of genotypes, namely, GG, GA, and AA for the entire data population. Individuals with genotypes that have the same allele pairs are called homozygotes, while differing allele pairs are called heterozygotes.

### Statistical analysis

Genotype and gene frequencies were calculated based on Pichner [11] using the formula 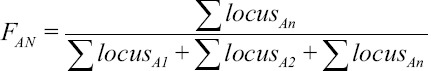, where F_An_ is frequency of gene A at the n^th^ locus, which were then used to test population equilibrium based on the Hardy-Weinberg (H-W) principle. Pearson’s Chi-square test was used to verify that the samples did not deviate from H-W equilibrium. The test statistics were computed using the formula 
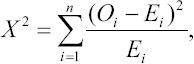
 where X^2^ represents the Chi-square value, O_i_ the observed frequency, E_i_ the expected frequency, and n the number of possible outcomes for each event [12].

### *PRL* gene polymorphism associated with reproductive traits

The mean and standard deviations of reproductive traits were calculated for each breed and genotype. Genotypic associations with reproductive traits were determined using one-way analysis of variance and the GLM procedure of SAS Institute (2001), according to the following model:

Y=μ + G + e

where Y=dependent variables, μ=population mean, G=genotypes’ effect on reproductive traits, and e=error term. If a genotype or haplotype was found to act significantly on reproductive traits, then a Tukey test was performed using a significance level of p<0.05 and p<0.01. Linear regression analyses were conducted to assess the linear trend of the reproductive traits across genotypes. The coefficient correlation (r) obtained was then compared with the standard grouping according to [13], that is, 0.00-0.10 (negligible), 0.10-0.39 (weak), 0.40-0.59 (moderate), 0.60-0.79 (strong), and 0.80-1.0 (very strong). The coefficient of determination of linear regression (r^2^) representing the variance proportion (%), accounted for by a genotype for each reproductive trait, was also computed. The genotypic value was determined based on its dominance: GG is the dominant homozygote with a value of 3, heterozygote GA with a value of 2, and a recessive homozygous AA with a value of 1.

## Results

### Quantitative traits

Based on the results from this study, the reproductive traits, that is, AFE, BW, FEW, and EP, obtained from 280 ILDs consisting of Tegal (F0) and Magelang (F0) ducks, and the reciprocal crosses, namely, Gallang (F1) and Maggal (F1), were relatively varied. The AFE of base (F0) and offspring generations (F1) differed significantly (p<0.01). FEW and EP traits were also significantly different (p<0.05). The BW of Tegal ducks (F0) differed significantly (p<0.01) from Magelang ducks (F0) and their crosses (F1). Magelang ducks tended to have higher means of reproductive traits compared to Tegal, Gallang, and Maggal ducks (Table-2).

**Table-2:** Mean and standard deviation of reproductive traits for Tegal, Magelang, and its reciprocal crosses (Gallang and Maggal).

ILD^1^	Mean and standard deviation of reproductive traits			

	AFE (d)	BW (g)	FEW (g)	EP (%)
Tegal (F0)	190.59^a^±6.28	1220.23^a^±106.05	61.46^a^±4.41	56.55^a^±7.31
Magelang (F0)	187.72^a^±3.96	1717.08^bc^±151.35	64.83^a^±4.57	74.12^a^±11.41
Gallang (F1)	146.51^b^±11.99	1637.01^cc^±113.26	55.94^ab^±6.97	68.36^ab^±12.95
Maggal (F1)	160.31^c^±15.93	1603.57^dc^±54.84	56.90^ab^±7.61	79.49^ab^±11.89

^a,b,c^=Values in each column with different superscripts show significant, ILD=Indonesian local ducks, AFE=Age at first egg, BW=Body weigh at first egg, FEW=First egg weight, EP=Egg production

### DNA amplification in the *PRL* gene region using PCR

**Figure-2:**
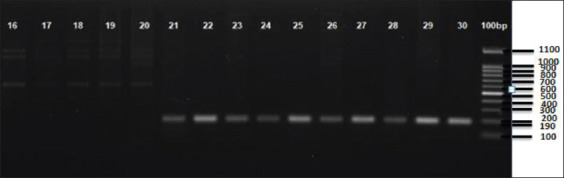
Polymerase chain reaction electrophoresis product of Prolactin Gene of Indonesian local ducks (190 bp) with PL-AnasPF L2376 and PL-AnasPR H2565 primer pairs using low melting agarose gel 1%.

A PCR product of 190 bp specific to the primer pair used was obtained after the optimization of the PCR process. The successful amplification was demonstrated by PCR fragments separated by electrophoresis in low melting agarose gel 1% and 1×TBE buffer in the Submarine Electrophoresis device (Hoefer, USA), at 50 V for 15 min (Figure-2).

### Identification of *PRL* gene polymorphisms in ILD

**Figure-3:**
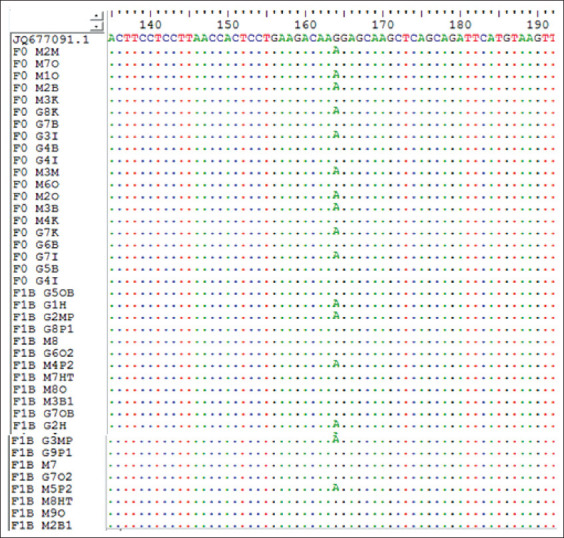
The results of sequence alignment of prolactin gene at position 164 nt of Indonesian Local Ducks. A=Adenine, T=Thymine, G=Guanine and C=Cytosine.

**Figure-4:**
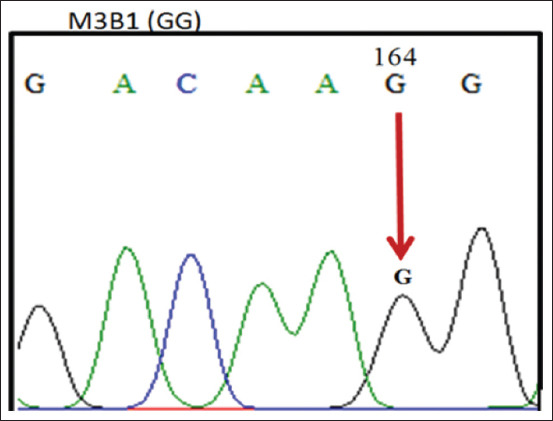
The GG Genotype.

**Figure-5:**
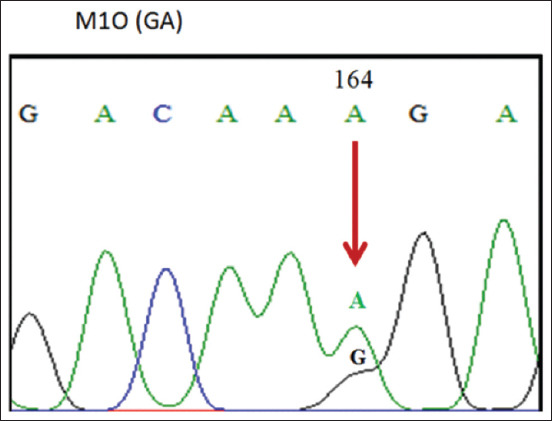
The GA Genotype.

**Figure-6:**
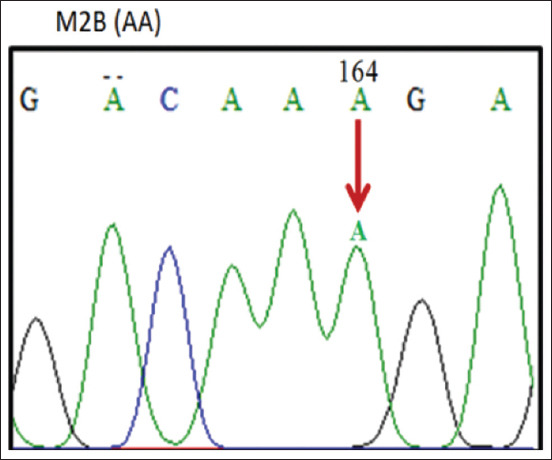
The AA Genotype.

The 190 bp PCR products of the *PRL* gene of ILDs were aligned with data obtained from GenBank (JQ677091.1), using the ClustalW and BioEdit programs (https://bioedit.software.informer.com/7.2/) and found mutations at position 164 nt. The sequencing alignment (Figure-3) identified three SNPs, namely, c.164G >G, c.164G >A, and c.164A >A. A mutation of guanine (G) to adenine (A) indicated the nucleotide variation in the region. Three genotypes were obtained, namely, GG (homozygote) (Figure-4), GA (heterozygote) (Figure-5), and AA (homozygote) (Figure-6).

Figure-4 shows that the SNPs contained in the samples and GenBank data (JQ677091.1) are the same, which means there are no mutations indicating a GG (homozygous) genotype by 1 (one) peak of the guanine electropherogram (G). Figure-5 shows the occurrence of an imperfect or partial mutation of the guanine base (G) to adenine (A) so that 2 (two) peaks of the G and A electropherograms occur and a GA (heterozygote) genotype is obtained. Figure-6 shows the complete mutation from guanine (G) to adenine (A) so that 1 (one) peak of the electropherogram A appears and an AA (homozygous) genotype is obtained.

### Chi-square test

Based on the identification of the *PRL* gene polymorphism at the 164 nt locus in ILD, the genotype and allele frequencies can then be determined. Using the genotyping information, Person’s Chi-square (χ^2^) test was used to test for H-W equilibrium in ILD populations. The results of the Chi-square test are presented in Table-3. The GG, GA, and AA genotypes did not conform to H-W equilibrium in any of the Tegal and Magelang (F0), and Gallang (F1) duck populations. In contrast, the Maggal duck (F1) population was found to be in equilibrium.

**Table-3:** Chi-square test (χ^2^) of locus prolactin gene in Indonesian local duck populations.

ILD^1^	Item	Genotypes			Alleles		χ^2^ value
	
		GG	GA	AA	G	A	
Tegal (F0)	Number of population	28	14	28			
	Genotypic frequency	0.40	0.20	0.40	0.50	0.50	25.20
	Expected frequency	0.25	0.50	0.25			
Magelang (F0)	Number of population	42	0	28			
	Genotypic frequency	0.60	0	0.40	0.60	0.40	70
	Expected frequency	0.36	0.48	0.16			
Gallang (F1)	Number of population	42	14	14			
	Genotypic frequency	0.60	0.20	0.20	0.70	0.30	19.21
	Expected frequency	0.49	0.42	0.09			
Maggal (F1)	Number of population	56	14	0			
	Genotypic frequency	0.80	0.20	0	0.90	0.10	−0.99
	Expected frequency	0.81	0.18	0.01			

χ^2^_0.01, 2_=9.21, ^1^ILD=Indonesian local duck

### SNP amino acid changes

Variation in the *PRL* gene fragment was found at SNP c.164G >A, which caused amino acid changes from position 121 nt (JQ677091.1), to 116-117 nt (in Tegal, Magelang, Gallang, and Maggal ducks). Changes in position, weight, number, and percentage of amino acid molecules of the *PRL* gene in Tegal, Magelang (F0), Gallang, and Maggal (F1) ducks are presented in Table-4.

**Table-4:** Changes in position, weight, number, and percentage of amino acid molecules of prolactin gene in Tegal, Magelang (F0), Gallang, and Maggal (F1) ducks.

Amino acid	JQ677091.1^1^		Tegal (F0)						Magelang (F0)				Gallang (F1)						Maggal (F1)			
			
			GG^2^		GA^2^		AA^2^		GG^2^		AA^2^		GG^2^		GA^2^		AA^2^		GG^2^		GA^2^	
Position MW^3^	121 9867.47		116 9526.14		116 9540.16		116 9540.16		116 9540.16		116 9540.16		116 9572.23		116 9540.16		117 9597.21		116 9526.14		116 9540.16	

	N^4^	Mol%	N	Mol%	N	Mol%	N	Mol%	N	Mol%	N	Mol%	N	Mol%	N	Mol%	N	Mol%	N	Mol%	N	Mol%

Ala	37	30.58	33	28.45	34	29.31	34	29.31	33	28.45	34	29.31	33	28.45	34	29.31	34	29.06	33	28.45	34	29.31
Cys	24	19.83	24	20.69	24	20.69	24	20.69	24	20.69	24	20.69	25	21.55	24	20.69	24	20.51	24	20.69	24	20.69
Asp	0	0.00	0	0.00	0	0.00	0	0.00	0	0.00	0	0.00	0	0.00	0	0.00	0	0.00	0	0.00	0	0.00
Glu	0	0.00	0	0.00	0	0.00	0	0.00	0	0.00	0	0.00	0	0.00	0	0.00	0	0.00	0	0.00	0	0.00
Phe	0	0.00	0	0.00	0	0.00	0	0.00	0	0.00	0	0.00	0	0.00	0	0.00	0	0.00	0	0.00	0	0.00
Gly	30	24.79	29	25.00	28	24.14	28	24.14	29	25.00	28	24.14	28	24.14	28	24.14	29	24.79	29	25.00	28	24.14
His	0	0.00	0	0.00	0	0.00	0	0.00	0	0.00	0	0.00	0	0.00	0	0.00	0	0.00	0	0.00	0	0.00
Ile	0	0.00	0	0.00	0	0.00	0	0.00	0	0.00	0	0.00	0	0.00	0	0.00	0	0.00	0	0.00	0	0.00
Lys	0	0.00	0	0.00	0	0.00	0	0.00	0	0.00	0	0.00	0	0.00	0	0.00	0	0.00	0	0.00	0	0.00
Leu	0	0.00	0	0.00	0	0.00	0	0.00	0	0.00	0	0.00	0	0.00	0	0.00	0	0.00	0	0.00	0	0.00
Met	0	0.00	0	0.00	0	0.00	0	0.00	0	0.00	0	0.00	0	0.00	0	0.00	0	0.00	0	0.00	0	0.00
Asn	0	0.00	0	0.00	0	0.00	0	0.00	0	0.00	0	0.00	0	0.00	0	0.00	0	0.00	0	0.00	0	0.00
Pro	0	0.00	0	0.00	0	0.00	0	0.00	0	0.00	0	0.00	0	0.00	0	0.00	0	0.00	0	0.00	0	0.00
Gln	0	0.00	0	0.00	0	0.00	0	0.00	0	0.00	0	0.00	0	0.00	0	0.00	0	0.00	0	0.00	0	0.00
Arg	0	0.00	0	0.00	0	0.00	0	0.00	0	0.00	0	0.00	0	0.00	0	0.00	0	0.00	0	0.00	0	0.00
Ser	0	0.00	0	0.00	0	0.00	0	0.00	0	0.00	0	0.00	0	0.00	0	0.00	0	0.00	0	0.00	0	0.00
Thr	30	24.79	30	25.86	30	25.86	30	25.86	30	25.86	30	25.86	30	25.86	30	25.86	30	25.64	30	25.86	30	25.86
Val	0	0.00	0	0.00	0	0.00	0	0.00	0	0.00	0	0.00	0	0.00	0	0.00	0	0.00	0	0.00	0	0.00
Trp	0	0.00	0	0.00	0	0.00	0	0.00	0	0.00	0	0.00	0	0.00	0	0.00	0	0.00	0	0.00	0	0.00
Tyr	0	0.00	0	0.00	0	0.00	0	0.00	0	0.00	0	0.00	0	0.00	0	0.00	0	0.00	0	0.00	0	0.00

1 From Gene Bank,

2 Genotype,

3 MW=Molecular Weight (Dalton),

4 n=Number

### *PRL* gene polymorphisms associated with reproduction traits

The results of the association analysis of the *PRL* gene and reproductive traits are presented in Table-5. An association was identified between the genotypes of the *PRL* gene and reproductive traits (BW, FEW, and EP). There was a significant correlation (p<0.05), with a positive correlation coefficient of 0.10, between GG, GA, and AA genotypes with BW traits in Tegal and Magelang duck populations (F0), as well as between the GG and GA genotypes in Gallang duck populations (F1). A significant association (p<0.05) was also observed between the GG, GA, and AA genotypes with the FEW trait in the Gallang duck population (F1), although with a negative correlation coefficient (−0.198). A highly significant association (p<0.01) with a positive correlation coefficient of 0.14 was observed between the GG, GA, and AA genotypes of the *PRL* gene related to the EP trait in Magelang (F0), Gallang (F1), and Maggal (F1) populations. Conversely, there was no significant association (p>0.05) (with a negative correlation coefficient of −0.22) between the GG, GA, and AA genotypes of the *PRL* gene related to the AFE trait in each ILD population.

**Table-5:** Association of genotypes of prolactin gene with reproduction traits and their correlation coefficients on Tegal (F0), Magelang (F0), Gallang (F1), and Maggal (F1).

ILD^1^	Genotypes (G_n_)^2^	N^3^	Reproductive traits (mean and standard deviation)			

			AFE (d)	BW (g)	FEW(g)	EP (%)
Tegal (F0)	GG	28	188.93±4.54	1192.89^a^±101.93	62.54±4.73	55.99^a^±7.43
	GA	14	191.43±6.61	1187.14^ac^±72.33	60.29±3.81	61.83^ab^±6.32
	AA	28	191.82±7.38	1264.12^bc^±11.77	60.96±4.27	52.67^ac^±5.04
Magelang (F0)	GG	42	187.33±3.23	1688.5^a^±151.79	64.21±4.89	80.79^a^±7.57
	AA	28	188.32±4.85	1745.67^b^±147.15	65.25±4.07	64.10^b^±8.52
Gallang (F1)	GG	42	148.31±9.12	1698.24^a^±62.20	54.26^a^±6.35	62.42^a^±9.98
	GA	14	139.36±9.12	1620.57^b^± 85.45	55.43^a^±7.37	85.74^b^±8.15
	AA	14	148.29±11.79	1469.79^c^±77.89	61.5^b^±5.8	72.23^c^±8.76
Maggal (F1)	GG	56	160.38±16.73	1602.25±59.29	55.88^a^±7.48	75.49^a^±9.69
	GA	14	160.07±12.78	1608.86±32.39	61.67^b^±6.58	95.52^b^±2.89
r_GT_	-	-	−0.22	0.10	−0.198	0.14
r^2^_GT_ (%)	-	-	4.98	0.97	3.93	1.88

**Figure-7:**
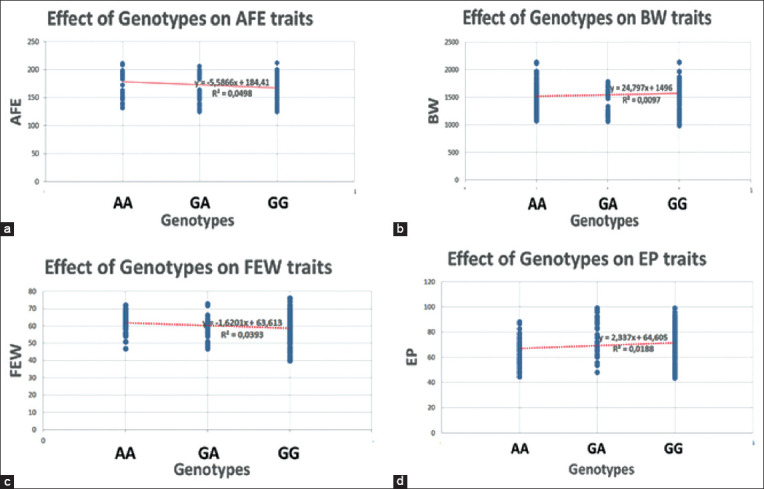
Genotypes effects of prolactin gene on reproductive traits of Indonesian Local Ducks on (a) AGE on first egg, (b) body weight at first egg, (c) first egg weight, and (d) egg production.

1 ILD=Indonesian Local Ducks,

2 G_n_=Genotypes,

3 N=Number of population. AFE=Age at First Egg; BW=Body Weigh at first egg; FEW=First Egg Weight; EP=Egg Production. ^a,b,c^=Values with different superscripts at the same column of the same ILD shows significant, r_GT_=Correlation coefficient of Genotype (G_n_) dan Traits (T), r^2^_GT_ (%)=Coefficients of determination of Genotype (G_n_) dan Traits (T) (Figure-7c) and EP (Figure-7d), respectively

### Genotypic effects of the *PRL* gene on reproductive traits

In this study, the genotypic effect of the *PRL* gene on reproductive traits is presented in Figure-7. The effect of this genotype indicates to what extent the *PRL* gene affects the AFE, BW, FEW, and EP traits. The regression analysis indicated that the *PRL* gene affected 4.98, 0.97, 3.93, and 1.88 % of the variation in AFE (Figure-7a), BW (Figure-7b), and FEW (Figure-7c) and EP (Figure-7d), respectively.

## Discussion

### Quantitative traits

The reproductive trait is an expression of quantitative traits, of which the appearance depends on genetic and environmental factors. In this study, supporting variables such as housing, initial age of study ducks, feeding (Table-1), and rearing management are relatively uniform and homogeneous so that the diversity of reproductive traits (AFE, BW, FEW, and EP) between Tegal (F0) and Magelang (F0) ducks and their reciprocal crosses, that is, Gallang (F1) and Maggal (F1) (Table-2), can be attributed to the genetic effects of the *PRL* gene (Table-5). The level of plasma *PRL* protein expression is critical in regulating the reproductive ability of poultry, including incubation and EP. In poultry, changes in plasma *PRL* levels are related to the expression of *PRL* mRNA in the anterior pituitary [14].

### Polymorphism and SNP genotyping of the *PRL* gene in ILD

As demonstrated in Figures-4-6, these base changes are one type of transition mutation. Changes in one purine base (G) to another purine base (A), or a change in one pyrimidine base (C) to another pyrimidine base (T) are called a transition mutation [15]. A study on Chinese ducks detected various polymorphisms (A-412G) in intron 1 of the *PRL* gene, with three genotypes, that is, AA, AG, and GG [16]. Two mutations occur in the non-coding area of intron 4 in g.3941T >G, and g.3975C >A. The analysis results indicated that each SNP is associated with reproductive traits in at least one duck [17]. The *PRL* gene sequence in adult ducks is similar to the amino acid sequences of *PRL* in chicken (93.4%), turkey (91.3%), and quail (91.3%) [18]. *PRL* gene polymorphisms relating to production traits have also been reported in chickens [18-21] and geese [22]. The fact that three SNPs produce three genotypes in the experimental population shows an association of polymorphic SNP heredity. Two *PR*L gene SNPs exist, namely, C-2161G and C-2402T in Fars native chickens of Iran [23].

### Population equilibrium

The Chi-square calculated value (Table-3) was greater than the Chi-square table with the corresponding degree of freedom. This suggests that the *PRL* gene distribution in Tegal (F0), Magelang (F0), and Gallang (F1) duck populations is not in H-W equilibrium. However, Maggal ducks exhibited H-W equilibrium for the *PRL* gene. The different H-W equilibrium states in this study population provide support that the frequency of alleles and genotypes in the duck population is relatively diverse. This further indicates that the population underwent selection, mutation, migration, or non-random mating within the population [4,24,25]. Thus, if a controlled selection is applied to the population it will provide a realistic selection response. H-W equilibrium in the population is attained if the frequency of alleles and genotypes remains constant from one generation to the next, as long as there is no selection, mutation, or migration and that random mating occurs [26,27].

### SNP amino acid changes

Table-4 demonstrates that the changes in amino acid position caused changes in the molecular weight, number, and percentage of amino acid molecules of the *PRL* gene in Tegal (F0), Magelang (F0), Gallang, and Maggal (F1) ducks. Changes occurred in the alanine, cysteine, glycine, and threonine amino acids. Amino acids containing the SNP c.376A >G and c.409G >A from the Melanocortin 1 Receptor **(***MC1R*) gene in Magelang ducks were altered. The mutation changed amino acid isoleucine to valine and valine/isoleucine (c.376A>G) and alanine to threonine and threonine/alanine (c.409A>G) [28]. Amino acid changes of the SNP c.700T >C and c.701G >A from the FSH gene at 234 nt were as follows: Tryptophan (Trp/W) to glutamine (Gln/Q) with changes in UGA amino acids to CAA (homozygotes), and Trp/W to arginine (Arg/R) with the change of UGA amino acids to CGA (heterozygotes) [29].

### *PRL* gene associations with reproductive traits

As demonstrated in Table-5, there was no association (p>0.05) between the GG, GA, and AA genotypes of the *PRL* gene with the AFE trait in any ILD population. However, there was a significant association (p<0.05) with BW and FEW traits. The association with the EP trait was highly significant (p<0.01). The highest EP in Tegal (F0), Gallang (F1), and Maggal (F1) ducks was obtained with the heterozygote genotype (GA), whereas the highest EP in Magelang duck (F0) was obtained with the dominant homozygote genotype (GG). Thus, it can be stated that the G gene is dominant for high EP and is associated with the relatively varied Quantitative Trait Locus in ILDs. This suggests that G gene population enrichment using molecular selection is useful for increasing the EP of ILD. Chinese local ducks carrying the GG genotype exhibited significantly higher EP and egg weights than those with the AG and AA genotypes (p<0.05) [16].

The genotypic value of the *PRL* gene positively correlates with the BW and EP traits, but has a negative relationship with the AFE and FEW traits (Figure-7). Correlation coefficients of 0.00-0.10 are considered a negligible correlation criterion, whereas correlation coefficients of 0.10-0.39 are weak correlations. The value of the correlation coefficient was small (non-significant), though it does not mean that the two variables are not associated. The two variables might have a strong relationship, but the correlation coefficient could be close to zero [13]. The relationship between two variables depends on the species, pairing traits, and the magnitude of the correlation coefficient [30]. Positive associations between the BW and EP traits, and genotype, indicate an increase in BW and EP traits for each increase in the number of dominant alleles (G), while a negative relationship indicates that an increase in the number of dominant alleles (G) is not followed by an increase in AFE and FEW traits. AFEs is an important trait that indicates sexual maturation and EP performance, which was negatively correlated with number of eggs laid [31,32].

### Genotypic effects of the *PRL* gene on reproductive traits

The effect of the *PRL* gene on reproductive traits is presented in Figure-7. The effect of the *PRL* gene genotype on AFE was 4.98 % (Figure-7a); BW 0.97 % (Figure-7b); FEW 3.93 % (Figure-7c); and EP 1.88 % (Figure-7d). This relatively small effect shows that many other factors influence AFE, BW, FEW, and EP traits. The influencing factors could be both exogenous (environmental) and endogenous (genetic) factors. The production and reproduction traits are controlled by polygenes [32]. Some genetic factors affected reproduction and production traits, besides the *PRL* gene. The FSH gene is reported to affect reproduction and production traits in ILDs [29]. In addition, reproduction and production traits in ducks are also affected by the type or species of livestock [30]. Some studies in Chinese local duck (Shaoxing) have reported that the melatonin receptor genes affect the age of first egg [33].

The correlation coefficient obtained can be used as the basis for a selection program. Genetic polymorphisms that have an effect on body weight or the production trait that plays a role in other economic improvements can be used as a selection tool [34]. Although the obtained correlation coefficient between the *PRL* gene and EP trait between the GG, GA, and AA genotypes in Tegal (F0), Magelang (F0), Gallang (F1), and Maggal (F1) duck populations was relatively small (0.14), it still had a significant positive (p<0.01) effect (Table-4). In a pig population being studied, the genotype frequency of the *PRL* receptor (*PRLR*) genotypes AA, AB, and BB was 0.247, 0.386, and 0.367, respectively. Pigs that carry the AA genotype had significantly higher numbers of piglets (p≤0.01) than those who had the AB or BB genotypes [35]. EP in poultry, among others, is influenced by the breed, body weight, and early egg lying age [10]. A significant difference (p>0.05) was found between genotypes (AA, AB, and BB) and the EP trait related to the *PRL* gene in Wan-xi White (China) and Rhine (European) goose breeds. Geese with the AA genotype laid more eggs than the AB or BB genotypes [22]. There was a significant relationship (p<0.01) between polymorphic nucleotides at position C1715301T in VIPR1 (VIPR1/TaqI) with the number of eggs laid (p<0.05) in Lien Minh chickens [20]. The genes that had a significant effect on EP among others, are the *PRL* gene and the *PRLR* gene [6,36].

The action of *PRL* is biologically mediated by the *PRLR*, which has structural similarities with the *PRL* Growth Hormone Receptor and belongs to the Class I cytokine receptor superfamily. A sharp increase of plasma *PRL* levels can cause a decrease in EP [37]. In some species, the anti-gonadal effects of *PRL* can cause inhibition of follicle formation and inhibited egg lying. Potential anti-gonadal effects of *PRL* through inhibition of Gonadotropin-releasing hormone I and II and Luteinizing Hormone have been demonstrated in *in vitro* testing in poultry [38,39] and is supported by evidence of the anti-gonadal effects of *PRL* in *in* *vivo* testing of several species [37]. However, the anti-gonadal effects of *PRL* have not been found in other species [40,41]. The production and reproduction traits with estimated heritability of low to moderate from 0.13 to 0.20 [42,43] make conventional breeding methods ineffective. Therefore, molecular-aided selection is a powerful tool for improving the egg-production related traits and increasing economic benefits.

The *PRL* gene is polymorphic and is an important marker, and can be used to increase the economic characteristics of local ducks [44]. Genetic variations in the *PRL* genes of domestic poultry can be used as genetic markers for further selection. Sequences of the *PRL* gene in various bird species can provide useful information to uncover the physiological and general functions as well as species-specific mechanisms for gene expression. The use of genetic markers has the potential to increase selection intensity and is supported to be most effective way of selection [45].

## Conclusion

Based on the results of this study, it can be concluded that SNP genotypes of the *PRL* gene are polymorphic and associate with positive genetic effects in reproductive traits, especially EP in ILDs. The GA genotype obtained (SNP c.164G >A) can be used as a marker-assisted selection candidate for the high EP trait.

## Authors’ Contributions

DP compiled the research ideas, designed the main framework, and composed the manuscript. RSSS, SAS, AS, and DPC contributed in coordinating field data collections. SAS conducted the statistical analysis. AS compiled and revised the manuscript. II read, criticized, and revised the text according to its scientific content. All authors have read and agreed to the final draft.
